# Prevalence and determinants of atrial fibrillation progression in paroxysmal atrial fibrillation

**DOI:** 10.1136/heartjnl-2022-321027

**Published:** 2022-07-20

**Authors:** Bao-Oanh Nguyen, Vanessa Weberndorfer, Harry JGM Crijns, Bastiaan Geelhoed, Hugo Ten Cate, Henri Spronk, Abraham Kroon, Ruben De With, Meelad Al-Jazairi, Alexander H Maass, Yuri Blaauw, Robert G Tieleman, Martin E W Hemels, Justin Luermans, Joris de Groot, Cornelis P Allaart, Arif Elvan, Mirko De Melis, Coert Scheerder, Anton Jan van Zonneveld, Ulrich Schotten, Dominik Linz, Isabelle Van Gelder, Michiel Rienstra

**Affiliations:** 1 Cardiology, University Medical Centre Groningen Thoraxcentre, Groningen, The Netherlands; 2 Cardiovascular Research Institute Maastricht (CARIM), Maastricht Universitair Medisch Centrum+, Maastricht, The Netherlands; 3 Department of Internal Medicine, Maastricht Universitair Medisch Centrum+, Maastricht, The Netherlands; 4 Cardiology, Martini Hospital, Groningen, The Netherlands; 5 Department of Cardiology, Rijnstate Hospital, Arnhem, The Netherlands; 6 Department of Cardiology, Radboud University Nijmegen, Nijmegen, The Netherlands; 7 Department of Cardiology, Amsterdam UMC Locatie AMC, Amsterdam, The Netherlands; 8 Department of Cardiology, Amsterdam UMC Locatie VUmc, Amsterdam, The Netherlands; 9 Cardiology, Isala Zwolle, Zwolle, The Netherlands; 10 Medtronic Bakken Research Centre, Maastricht, The Netherlands; 11 Department of Internal Medicine (Nephrology) and the Einthoven Laboratory for Experimental Vascular Medicine, Leiden Universitair Medisch Centrum, Leiden, The Netherlands; 12 Physiology, Maastricht Universitair Medisch Centrum+, Maastricht, The Netherlands

**Keywords:** atrial fibrillation, biomarkers, risk factors

## Abstract

**Objective:**

Atrial fibrillation (AF) often progresses from paroxysmal AF (PAF) to more permanent forms. To improve personalised medicine, we aim to develop a new AF progression risk prediction model in patients with PAF.

**Methods:**

In this interim-analysis of the Reappraisal of AF: Interaction Between HyperCoagulability, Electrical Remodelling, and Vascular Destabilisation in the Progression of AF study, patients with PAF undergoing extensive phenotyping at baseline and continuous rhythm monitoring during follow-up of ≥1 year were analysed. AF progression was defined as (1) progression to persistent or permanent AF or (2) progression of PAF with >3% burden increase. Multivariable analysis was done to identify predictors of AF progression.

**Results:**

Mean age was 65 (58–71) years, 179 (43%) were female. Follow-up was 2.2 (1.6–2.8) years, 51 of 417 patients (5.5%/year) showed AF progression. Multivariable analysis identified, PR interval, impaired left atrial function, mitral valve regurgitation and waist circumference to be associated with AF progression. Adding blood biomarkers improved the model (C-statistic from 0.709 to 0.830) and showed male sex, lower levels of factor XIIa:C1-esterase inhibitor and tissue factor pathway inhibitor, and higher levels of N-terminal pro-brain natriuretic peptide, proprotein convertase subtilisin/kexin type 9 and peptidoglycan recognition protein 1 were associated with AF progression.

**Conclusion:**

In patients with PAF, AF progression occurred in 5.5%/year. Predictors for progression included markers for atrial remodelling, sex, mitral valve regurgitation, waist circumference and biomarkers associated with coagulation, inflammation, cardiomyocyte stretch and atherosclerosis. These prediction models may help to determine risk of AF progression and treatment targets, but validation is needed.

**Trial registration number:**

NCT02726698.

**WHAT IS ALREADY KNOWN ON THIS TOPIC**

- Atrial fibrillation progression is associated with adverse cardiovascular outcome.
- The rate of atrial fibrillation progression varies and depends among others on type of rhythm monitoring.
- Predictors of atrial fibrillation progression have not been well established with long-term continuous rhythm monitoring.

**WHAT THIS STUDY ADDS**

- This study develops an atrial fibrillation progression risk prediction model and elucidates underlying pathophysiological mechanisms in comprehensively phenotyped patients with paroxysmal atrial fibrillation using long-term continuous rhythm monitoring.
- Our clinical multivariate model had a C-statistic of 0.709.
- The addition of the blood biomarkers improved the initial model to a C-statistic of 0.830.
- We found that predictors for progression were multifactorial including atrial remodelling, sex, mitral valve regurgitation, waist circumference and blood biomarkers associated with coagulation, cardiac stretch, cholesterol metabolism, inflammation and the immune system.
- Validation is needed before implementation into clinical practice.

**HOW THIS STUDY MIGHT AFFECT RESEARCH, PRACTICE OR POLICY**

- This model could be clinically useful, and serves to enhance knowledge on underlying mechanisms causing progression of atrial fibrillation.
- In combination with extensive phenotyping, our prediction model gives a more in-depth view into predicting risk factors for atrial fibrillation progression.
- Continuous rhythm monitoring provides a more detailed and accurate view into atrial fibrillation progression.

## Introduction

Atrial fibrillation (AF) is a progressive disease, usually starting with self-terminating short-lasting paroxysmal episodes that often progresses to more frequent episodes, eventually leading to long-lasting non-self-terminating persistent and permanent AF.^1^ Progression of AF has been associated with an increased risk of cardiovascular morbidity and mortality and reduces the efficacy of pharmacological and interventional rhythm control strategies.^2 3^ AF progression rates vary between studies because of differences in duration of follow-up, in comprehensive phenotyping of patients and strategies of rhythm monitoring.^3–5^

Appropriate treatment of risk factors can improve sinus rhythm maintenance, cardiovascular outcome and reverse AF progression.^6 7^ The most established risk factors for AF progression are age, hypertension, obesity, heart failure and diabetes.^5 8^ Interestingly, hypercoagulability may be involved in increasing the risk of stroke and in AF progression.^9^ A detailed and multimodal phenotyping at baseline and continuous rhythm monitoring has potential to increase our knowledge of AF progression and in turn contribute to personalised medicine.^2 10^

Therefore, the aim of the Reappraisal of AF: Interaction Between HyperCoagulability, Electrical Remodelling, and Vascular Destabilisation in the Progression of AF (RACE V) study is to develop a clinical AF progression risk prediction model using extensive phenotyping and continuous rhythm monitoring in patients with paroxysmal AF (PAF). In addition, to improve the clinical model and elucidate underlying pathophysiological mechanisms of AF progression, we included blood biomarkers in the progression risk prediction model.

## Methods

### Study design

The RACE V study has previously been described.^11^ In brief, the RACE V study is a prospective, investigator-initiated, Dutch multicentre observational study (Clinicaltrials.gov identifier NCT02726698).

A detailed overview of inclusion and exclusion criteria is provided in online supplemental table S1. Briefly, the aim is to include 750 patients with a history of PAF <10 years. Eligible patients had ≥2 documented episodes of PAF or one documented episode combined with ≥2 symptomatic episodes suspected of being AF, were willing to undergo implantation of a Medtronic (Minneapolis, USA) Reveal LINQ^®^ implantable loop recorder, and did not have a history of persistent AF (intention to undergo), pulmonary vein isolation (PVI) or current amiodarone treatment. Patients with Medtronic pacemakers were also eligible if atrial high rate episodes >190 beats per min lasting >6 min, qualified as AF episodes, were detected. For the current analysis, we included patients that had ≥1 year of continuous rhythm monitoring as of 1 May 2020.

10.1136/heartjnl-2022-321027.supp1 Supplementary data

### Patient and public involvement

Patients and the public were not involved in the design or implementation of the study.

### Clinical assessment

At baseline, clinical history, symptomatology, current medication, physical examination and a 12-lead ECG were assessed. Additionally, echocardiography, vascular assessment and cardiac CT was done, processed and analysed in a central core lab (online supplemental figure S1, online supplemental data). In brief, in addition to the standard echocardiography measurements, strain measurements were performed in sinus rhythm using a point-and-click method to trace endocardial borders with a vendor-independent software (TOMTEC-ARENA, Imaging Systems, Germany). The cardiac CT was performed as a non-contrast ECG-gated scan to assess coronary calcium scores, epicardial and pericardial fat. Vascular assessment of the carotid arteries included measurements of intima-media thickness, pulse wave velocity and plaques.

**Figure 1:**
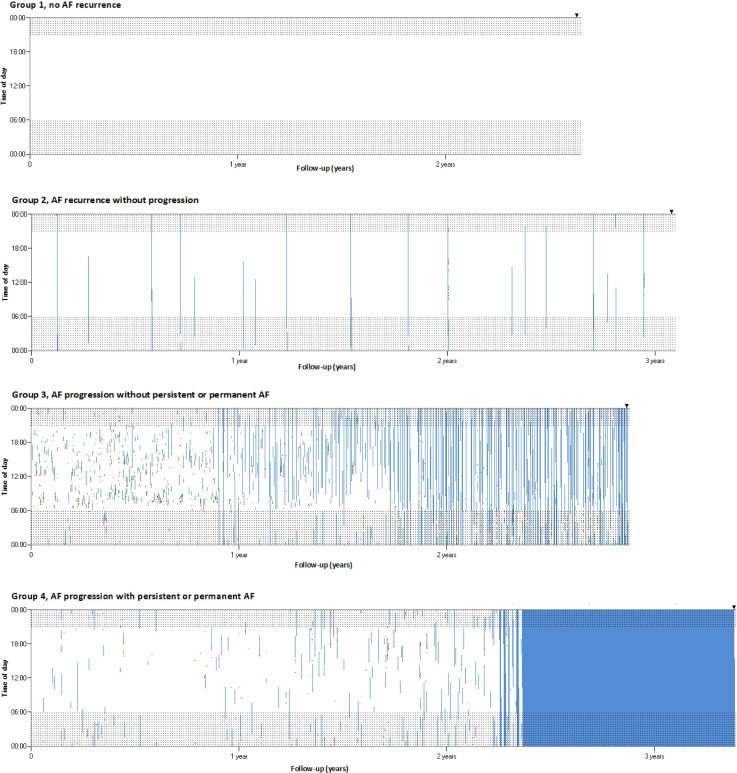
Examples of continuous rhythm monitoring. Examples of individual patients without AF progression (group 1 and group 2) and with AF progression (group 3 and 4) during follow-up. The X-axis presents follow-up in years, the Y-axis is the time of the day. Shaded areas indicate nightly hours. Black triangle presents day of end of analysis. White means no AF is present, and blue represents ongoing episodes of AF. AF initiations are shown in red and AF terminations are shown in green. AF, atrial fibrillation.

### Blood biomarkers

At baseline, peripheral blood samples were collected (only during sinus rhythm with interrupted anticoagulation). With multiplex immunoassays, 92 cardiovascular biomarkers from the Olink Cardiovascular III panel were assessed by Olink Bioscience (Uppsala, Sweden) in EDTA plasma baseline samples (online supplemental table S2). Complexes of activated coagulation enzymes (FXIIa, FXIa, FIXa, FXa and thrombin) with their corresponding natural inhibitors (antithrombin, alpha1-anti-trypsine or C1-esterase inhibitor) ELISA assays were performed to assess the degree coagulation activity in EDTA plasma and citrated plasma samples at baseline.^12^

### Follow-up

All patients were treated according to the European Society of Cardiology AF guidelines.^13^ Follow-up visits were performed at 1 and 2.5 years (online supplemental figure S1). Patients could consent for 2.5 years continuous rhythm monitoring, until end of battery of Reveal LINQ, or for 4 years in case patients had a pacemaker.

In order to collect continuous data on arrhythmias, all patients received a home monitoring device (Medtronic Carelink). Both Reveal LINQ and pacemaker were set to AT/AF detection settings (online supplemental data).

### Definition and outcome

The primary outcome was AF progression. Before assessing AF progression, all collected episodes were independently adjudicated and corrected by five physicians. Two methods were used to assess AF progression and compared. For the first method, all AF episodes were put into a custom-made software using Microsoft Visual Basic to visualise in a graphical overview all AF episodes per patient (figure 1), which was done by six physicians. Four groups were discerned: (1) no AF recurrences during follow-up; (2) recurrences of PAF without apparent increase in number and/or duration of AF episodes based on visual inspection; (3) recurrence of PAF with increase in number and/or duration of AF based on visual inspection, but without persistent or permanent AF; (4) development of persistent or permanent AF (figure 1).

For the second method, a mathematical formula (online supplemental data) was created using a weighed AF burden with AF episodes early during follow-up weighing less than AF episodes at the end of follow-up. AF burden was defined as the cumulative duration of all AF episodes from baseline onwards, divided by total duration of monitoring. For patients without successful PVI, a 90-day blanking period after PVI was applied.

The primary outcome was AF progression, defined as (1) development of persistent or permanent AF during follow-up or (2) an increase of >3% AF burden over the first 6 months or total follow-up. Duration of monitoring for current analysis lasted until 1 May 2020, until last available rhythm monitoring for patients that died after >1 year of continuous rhythm monitoring, until date of PVI, or in case of a successful PVI.

We considered the mathematical formula as leading of both methods, because it is easier to apply in other independent cohorts. Results from both methods were compared and showed that no patients classified as ‘without AF progression’ by physicians were ‘with AF progression’ according to the mathematical formula. Fourteen (3%) patients who were classified as AF progressors (from group 3) by physicians did not have AF progression according to the mathematical formula. These patients were eventually categorised as no AF progression.

### Statistical analysis

Baseline characteristics are presented as mean±SD for normally distributed data, and median and iIQRs for non-normally distributed continuous data. Categorical data are presented as numbers with percentages, biomarker multiplex immunoassay data as arbitrary units on a log2 scale. Fisher’s exact test was used for binary variables, and T-test or Wilcoxon rank-sum test was used for continuous variables. Collected baseline variables including core lab data, with p<0.10 in the age-adjusted and sex-adjusted logistic regression, with exception of European Heart Rhythm Association (EHRA) class, number of comorbidities, CHA_2_DS_2_-VASc score and medications, were included in a bidirectional stepwise variable selection leading to a final multivariable logistic regression model. Bidirectional stepping was done for model building and reduction, with a p value ≥0.05 as a criterion for removing a variable from the model (online supplemental data). Imputation was implemented for missing values using the R package mice. For each logistic regression, ‘massive imputation’ was performed, which means that all variables in a model were at the same time also used for the imputation needed for the fit of that model. For the second model, the Olink Cardiovascular III panel biomarkers (online supplemental table S6) and coagulation markers were added to the stepwise variable selection process if they reached p<0.10 in initial age-adjusted and sex-adjusted logistic regression to assess if the model would improve. Age and sex were forced into both multivariate models. Interactions between variables was tested, no significant interactions were found. The Harrell’s binary C-index was used for goodness-of-fit measure. P value <0.05 was considered statistically significant. Internal validation was done using bootstrapping. Analyses were conducted with R V.3.3.3 (www.r-project.org).

## Results

For the present analysis, we included 417 patients (table 1, online supplemental table S3). Median age was 65 (58–71) years, and 179 (43%) patients were women. Median follow-up of continuous rhythm monitoring was 2.2 (1.6–2.8) years. A total of 162 215 episodes were classified as AF by the automated algorithm, 53 397 (32.9%) were adjusted after adjudication, resulting in 119 120 remaining AF episodes (reasons for adjustments are presented in online supplemental data).

**Table 1:** Baseline characteristics

Characteristic	AF progression (n=51)	No AF progression (n=366)	Total population (n=417)	P value
Age (years)	64 (60–73)	65 (58–71)	65 (58–71)	0.278
Female sex	15 (29%)	164 (45%)	179 (43%)	0.049
Total history AF (years)	2.8 (0.9–4.9)	2.6 (0.7–5.2)	2.6 (0.7–5.1)	0.803
Heart failure	20 (39%)	104 (28%)	124 (29%)	0.274
HFrEF	4 (8%)	6 (2%)	10 (2%)	0.025
HFpEF	16 (31%)	98 (27%)	114 (27%)	1
Hypertension	46 (90%)	292 (80%)	338 (81%)	0.086
Diabetes mellitus	5 (10%)	29 (8%)	34 (8%)	0.59
Coronary artery disease	11 (22%)	37 (10%)	48 (12%)	0.031
Atherosclerosis*	26 (51%)	178 (49%)	204 (49%)	0.767
Peripheral artery disease	2 (4%)	1 (0%)	3 (1%)	0.041
Ischaemic stroke	1 (2%)	18 (5%)	19 (5%)	0.491
Pacemaker	9 (18%)	16 (4%)	25 (6%)	0.001
Number of comorbidities†	3 (2–4)	2 (2–3)	2 (2–3)	0.05
CHA_2_DS_2_-VASc score‡				0.016
<2	6 (12%)	101 (28%)	107 (26%)	
≥2	45 (88%)	265 (72%)	310 (74%)	
Physical examination				
Height (cm)	178 (170–185)	177 (169–184)	178 (169–184)	0.492
Weight (kg)	88 (73–102)	84 (74–96)	85 (74–97)	0.268
Body mass index (kg/m^2^)	27 (25–32)	27 (24–30)	27 (24–30)	0.708
Waist circumference (cm)	105 (99–113)	100 (92–108)	100 (93–108)	0.004
Laboratory results				
eGFR (mL/min/1.73 m^2^)	74 (67–86)	81 (70–90)	81 (69–90)	0.016
ECG				
PR interval	178 (160–199)	164 (149–184)	166 (150–186)	0.003
QRS interval	96 (90–106)	94 (86–103)	94 (88–104)	0.191
Medications				
β-Blocker	32 (63%)	181 (49%)	213 (51%)	0.099
Verapamil/Diltiazem	7 (14%)	66 (18%)	73 (18%)	0.557
Digoxin	2 (4%)	4 (1%)	6 (1%)	0.16
Class I antiarrhythmic drugs	5 (10%)	89 (24%)	94 (23%)	0.02
Class III antiarrhythmic drugs	3 (6%)	15 (4%)	18 (4%)	0.473
ACE inhibitor	11 (22%)	71 (19%)	82 (20%)	0.709
Angiotensin receptor blocker	14 (27%)	66 (18%)	80 (19%)	0.129
Statin	26 (51%)	119 (33%)	145 (35%)	0.012
Anticoagulant	45 (88%)	244 (67%)	289 (69%)	0.002
Vitamin K antagonist	10 (20%)	45 (12%)	55 (13%)	0.182
NOAC	35 (69%)	199 (54%)	234 (56%)	0.07
Echocardiographic variables				
Left atrial volume index (mL/m^2^)	34 (25–39)	29 (23–36)	29 (23–36)	0.038
Left atrial reservoir function (%)	31 (26–39)	37 (30–43)	36 (29–43)	0.045
Left atrial contractile function (%)	13 (11–17)	17 (13–22)	16 (13–21)	0.003
Left atrial conduction function (%)	18 (14–25)	19 (14–24)	19 (14–24)	0.965
Left ventricular ejection fraction (%)	50±8	51±8	51±8	0.893
Left ventricle strain	−14.2±2.5	−14.0±2.3	−14.0±2.4	0.76
Moderate aortic valve stenosis	0 (%)	3 (1%)	3 (1%)	1
Moderate aortic valve regurgitation	0 (%)	0 (%)	1 (0%)	1
Moderate mitral valve regurgitation	3 (6%)	4 (1%)	7 (2%)	0.045
CT				
Calcium score (Agatston)	131(5-492)	25 (0–228)	29 (0–275)	0.004
Pericardial fat	186 (148–235)	166 (121–231)	168 (124–233)	0.205
Epicardial fat	105 (77–130)	98 (71–128)	98 (72–128)	0.349
Vascular assessment				
IMT max-CCA >1 mm	19 (46%)	109 (34%)	128 (35%)	0.122
IMT max-all segments >1 mm	20 (49%)	154 (48%)	174 (48%)	1
Plaques	15 (29%)	125 (34%)	140 (34%)	0.407

Data are presented as mean±SD, number of patients (%) or median (IQR).

*Atherosclerosis is presence of history of myocardial infarction, percutaneous coronary intervention, coronary artery bypass graft, ischaemic cerebral infarction, peripheral vascular disease, Agatston score >400 or plaque.

†The number of comorbidities was calculated by awarding points for hypertension, heart failure, age >65 years, diabetes mellitus; coronary artery disease, BMI >25 kg/m^2^, moderate or severe mitral valve regurgitation and kidney dysfunction (eGFR <60).

‡The CHA_2_DS_2_-VASc score assesses thromboembolic risk. C=congestive heart failure/LV dysfunction, H=hypertension; A_2_=age ≥75 years; D=diabetes mellitus; S_2_=stroke/transient ischaemic attack/systemic embolism; V=vascular disease; A=age 65–74 years; Sc=sex category (female sex).

AF, atrial fibrillation; BMI, body mass index; CCA, common carotid artery; eGFR, estimated glomerular filtration rate; HFpEF, heart failure with preserved ejection fraction; HFrEF, heart failure with reduced ejection fraction; IMT, intima-media thickness; LV, left ventricular; NOAC, novel oral anticoagulation.

During follow-up, 48 (11.5%) patients showed no AF recurrences, and 318 (76.3%) had AF recurrences without AF progression. AF progression was seen in 51 (12.2%, 5.5% per year) patients: with an increase of >3% of AF burden but without deterioration into persistent or permanent AF in 16 (3.8%) patients, and with development of persistent or permanent AF in 35 (8.4%) patients (online supplemental table S4, figure 1).

Patients with AF progression were more often men, had more often coronary artery disease, larger waist circumference, longer PR interval, larger left atrial (LA) volume and reduced atrial contractile function (table 1).

**Figure 2:**
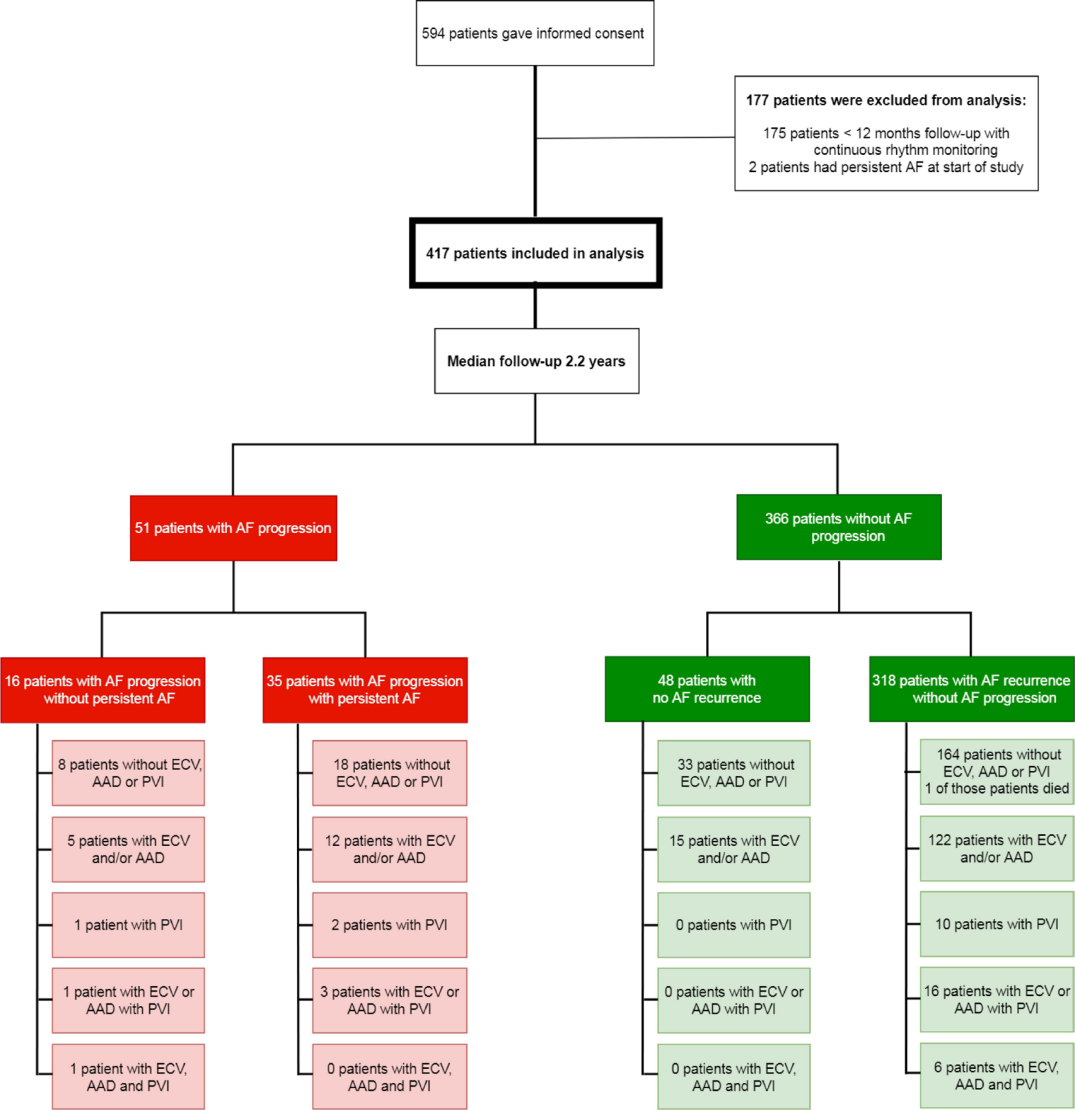
Flow chart of all patients. Four-hundred seventeen patients were included in current analysis. One patient died during follow-up and was included in the analysis until last rhythm monitoring date. AAD, anti-arrhythmic drugs; AF, atrial fibrillation; ECV, electrical cardioversion; PVI, pulmonary vein isolation.

During follow-up up, one patient died of an unknown cause. Eighteen patients received a pacemaker, 1 due to AV block and 17 patients due to sick sinus syndrome. Figure 2 presents rhythm control therapy during follow-up. No differences were seen in rhythm control therapy at baseline, follow-up and end of analysis between the AF progression and no AF progression group (online supplemental figure S2).

### Blood biomarkers

The baseline levels of the 92 biomarkers are presented in online supplemental table S5). At baseline, a significant difference between the groups was observed for 14 biomarkers.

Baseline coagulation markers are presented in online supplemental table S6). At baseline, the levels of factor XIIa:C1-esterase inhibitor complex and factor XIIa:antithrombin were significantly lower in the AF progression group compared with those without AF progression.

### Prediction models

The logistical analysis adjusted for age and sex with clinical variables showed that 14 variables were associated with AF progression (online supplemental table S7). The clinical multivariable model showed that a longer PR interval, an impaired LA contractile function, moderate mitral valve regurgitation and a higher waist circumference were associated with higher risk of AF progression (table 2A), C-statistic is 0.709 (95% CI 0.614 to 0.799). The optimism caused by overfitting in the C-statistic was 3.03%.

**Table 2:** (A) Multivariable clinical predictors for AF progression

	OR	95% CI	P value
Male sex	1.8	0.87 to 3.51	0.116
PR interval (per SD)	1.5	1.14 to 2.06	0.004
Impaired left atrial contractile function (per SD)	1.8	1.16 to 2.69	0.008
Moderate mitral valve regurgitation	5.9	1.02 to 33.97	0.048
Waist circumference (per SD)	1.5	1.06 to 2.03	0.023

(B) Multivariable predictors of AF progression including blood biomarkers			
	OR	95% CI	P value
Male sex	3.5	1.65 to 7.41	0.001
PR interval (per SD)	1.6	1.21 to 2.21	0.002
Impaired left atrial contractile function (per SD)	1.7	1.05 to 2.70	0.031
Factor XIIa:C1-esterase inhibitor (below median)	2.7	1.26 to 5.56	0.01
TFPI decrease (per SD)	1.8	1.23 to 2.53	0.002
NTproBNP (per SD)	1.9	1.28 to 2.81	0.002
PCSK9 (per SD)	1.6	1.09 to 2.21	0.015
PGLYRP1 (per SD)	1.5	1.11 to 2.11	0.009

AF, atrial fibrillation; NTproBNP, N-terminal pro-brain natriuretic peptide; PCSK9, proprotein convertase subtilisin/kexin type 9; PGLYRP1, peptidoglycan recognition protein 1; TFPI, tissue factor pathway inhibitor.

**Figure 3:**
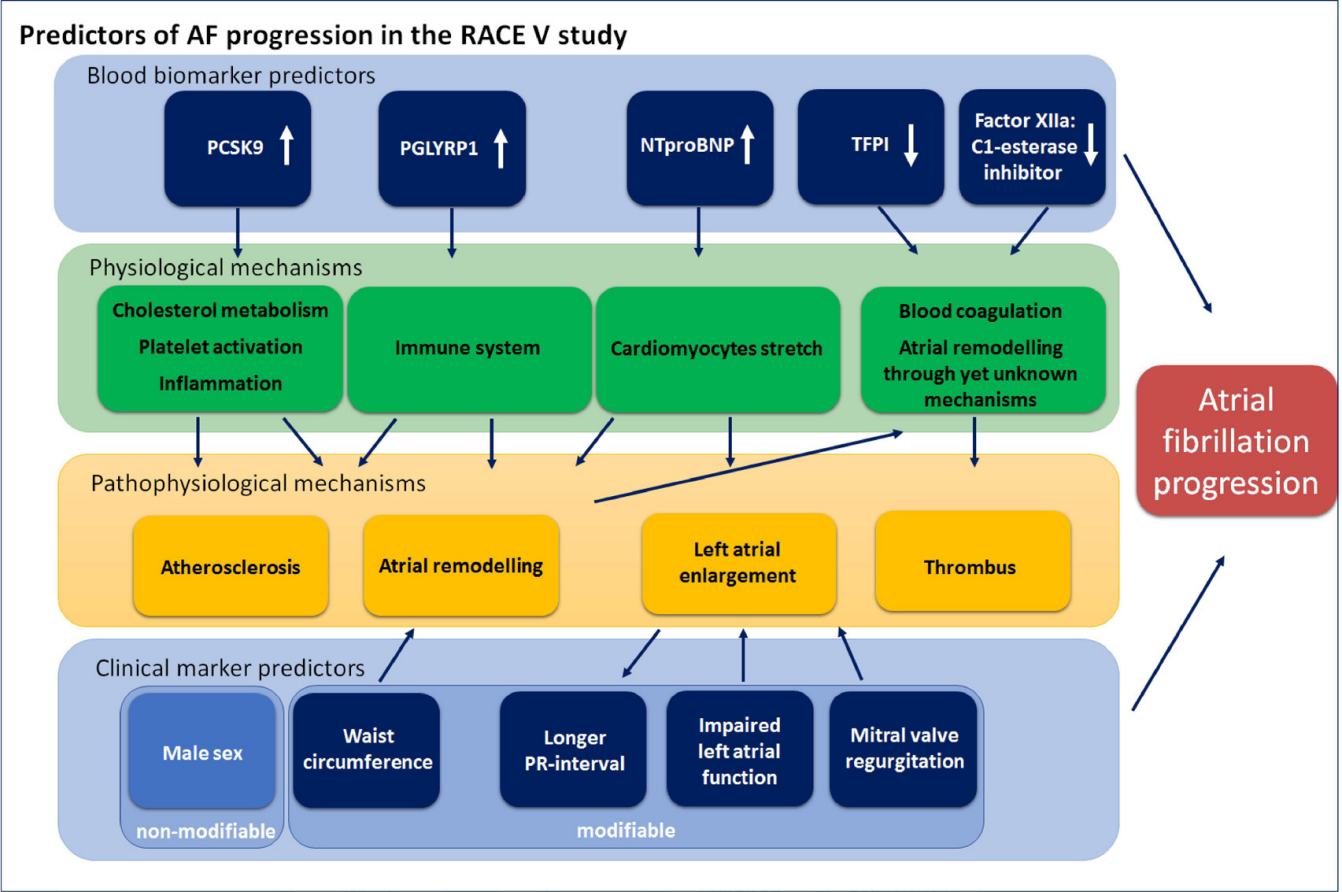
Predictors of atrial fibrillation (AF) progression in the Reappraisal of AF: Interaction Between HyperCoagulability, Electrical Remodelling, and Vascular Destabilisation in the Progression of AF study. Clinical markers and blood biomarkers as predictors for atrial fibrillation progression and their physiological and pathophysiological mechanisms. The blue boxes represent the multivariable predictors of atrial fibrillation progression. The green boxes represent the physiological mechanisms, the yellow boxes represent the pathophysiological mechanisms. NTproBNP, N-terminal pro-brain natriuretic peptide; PCSK9, proprotein convertase subtilisin/kexin type 9; PGLYRP1, peptidoglycan recognition protein 1; TFPI, tissue factor pathway inhibitor.

To improve the prediction model and to assess underlying pathophysiological mechanisms, an additional analysis including blood biomarkers was performed. The logistical analysis adjusted for age and sex showed 25 variables associated with AF progression (online supplemental table S8). Table 2B and figure 3 show the multivariable predictors of AF progression including blood biomarkers. The addition of the blood biomarkers improved the initial model (C-statistic 0.830 (95% CI 0.750 to 0.898)).

Based on the clinical multivariable model, a point risk score was developed for estimating an individual’s risk of AF progression at 2 years (table 3).

**Table 3:** Clinical point risk score

Sex	Points
Female	−2
Male	0
PR interval (ms)	Points
≤122	0
123–148	1
149–174	2
175–200	3
201–226	4
227–252	5
253–278	6
279–304	7
305–330	8
331–356	9
>356	10
Left atrial contractile function (%)	Points
≤12	3
13–17	2
18–22	1
23–27	0
28–32	-1
>32	-3
Waist circumference	Points
≤84	0
85–96	1
97–108	2
109–120	3
121–132	4
>132	5
Mitral valve regurgitation	Points
Yes	5
No	0

2-year risk estimation atrial fibrillation progression based on total points											
Total points	0	1	2	3	4	5	6	7	8	9	10
Risk %	2.3	3.1	4.2	5.6	7.5	9.9	13.0	16.9	21.6	27.2	33.7

AF, atrial fibrillation.

## Discussion

In the RACE V study, we assessed AF progression in comprehensively phenotyped patients with self-terminating PAF using long-term continuous rhythm monitoring. We showed that AF progression occurred in 5.5% of patients per year. Furthermore, using the clinical model markers of atrial remodelling, mitral valve regurgitation and waist circumference were associated with AF progression. The addition of blood biomarkers improved the C-statistic of the model and showed male sex, lower levels of coagulation markers and markers involved in cardiac stretch, cholesterol metabolism, inflammation and the immune system to be associated with AF progression.

Determining AF progression importantly depends on the type and amount of rhythm monitoring. Previous studies used limited rhythm monitoring and typically focused on progression from PAF to persistent or permanent AF.^3 4^ Yet, more studies suggest that increase of AF burden in PAF is also of importance.^14^ Therefore, we included increase of AF burden in our AF progression definition to avoid excluding patients with low burden that progressed to a significantly higher PAF burden. Although the majority of patients who showed AF progression deteriorated into persistent or permanent AF, 30% in the AF progression group were classified as progressors because of a likely clinically relevant increase of PAF burden.

In line with previous studies, we found multiple factors involved in AF progression associated with different underlying pathophysiological mechanisms.^3 8^ A longer PR interval and an impaired LA contractile function were associated with AF progression. Both can be seen as signs of more severe atrial structural remodelling (atrial cardiomyopathy) promoting AF progression.^15^ Previous studies showed that the PR interval was associated with incident AF but not with AF progression.^16^ Mitral valve regurgitation, well known to induce volume overload and LA enlargement and thus atrial remodelling, was also associated with AF progression.^17^ Atrial enlargement has been associated with incident and recurrent AF.^18^ Atrial contractility dysfunction has been related to duration of AF and may increase compliance of the atria, causing atrial cardiomyopathy, which is in turn associated with AF progression.^19^ A higher waist circumference was also associated with AF progression. A high body mass index (BMI) and obesity are well known risk factors for incident AF and AF progression.^5^ However, BMI does not take visceral fat distribution into account, which has been shown to be an independent marker for cardiovascular morbidities associated with AF and AF progression.^5^ Excess of visceral fat induces inflammation, which can promote atrial remodelling.^20^ Waist circumference could therefore be seen as a marker of visceral adipose tissue and thus being associated with AF progression.

In addition to a clinical prediction model, we sought to explore the underlying pathophysiological mechanisms for AF progression adding 101 blood biomarkers including coagulation markers to our analysis. The latter improved the prediction model significantly. Furthermore, it revealed that N-terminal pro-brain natriuretic peptide (NTproBNP) was a marker for risk of AF progression. NTproBNP is secreted by myocytes in response to multiple factors, including wall stress and is increased during AF, even without overt heart failure. Our results are comparable to previous studies showing that elevated NTproBNP levels are associated with incident AF and AF progression.^5 10 21^ Proprotein convertase subtilisin/kexin type 9 (PCSK9), also associated with AF progression in our model, is an enzyme involved in the homeostasis of cholesterol. Higher levels of PCSK9 are associated with cardiovascular events in patients with AF, possibly through atherosclerosis and inflammation.^22^ Peptidoglycan recognition protein 1 (PGLYRP1), a protein important in the innate immune response, was also associated with AF progression. PGLYRP1 is also involved in inflammation and associated with atherosclerosis. Elevated levels of PGLYRP1 have been associated with aortic wall thickness, aortic plaques and elevated Agatston scores.^23^ The fact that PCSK9 and PGLYRP1 were markers for AF progression suggests that vascular processes are of importance in AF progression.^24 25^

Lastly, lower levels of TFPI were associated with progression. This indicates that there is less inhibition of the extrinsic coagulation cascade in patients with AF progression due to reduced TFPI, resulting in increased activity of tissue factor and factor VIIa, and thus increased activation of the extrinsic coagulation pathway. Also, lower levels of factor XIIa:C1-esterase inhibitor, an enzyme inhibitor complex of the intrinsic coagulation cascade, were associated with AF progression. The origin of both and the role in AF progression remains unknown, but the postulated enhanced potential of tissue factor stimulated coagulation, due to lower TFPI activity, by itself would be in accordance with a role of hypercoagulability in driving AF as previously shown in preclinical studies.^9^ Recently, it was shown that duration of PAF was associated with higher levels of von Willebrand factor and factor VIII.^26^ Clearly, more research is warranted on the role of hypercoagulability in AF progression.

The model with additional biomarkers also revealed, unexpectedly, male sex as a clinical marker associated with AF progression. None of the previous studies showed sex differences involved in AF progression but data are still scare.^3 5 10^ In our study, the percentage of females was 43%, higher than in most studies. Interestingly, women with AF are usually older, having more comorbidities.^27 28^

In summary, our models, including the point risk score, may help to identify patients at risk for AF progression. It again emphasises that AF progression is a multifactorial disease and also suggests differences between sexes. The RACE V clinical risk score may contribute to determine individuals’ risks of AF progression and treatment targets. However, before introduction into clinical practice it first warrants validation. As a result, such a model may increase the complexity and burden for the physician. The Horizon 2020 EHRA-PATHs project aims to develop a software tool that may contribute to improve the feasibility of such a personalised therapeutic strategy.^29^

## Limitations

Our study has several limitations. First, in the RACE V study treatment was at the discretion of the treating physician, which may have influenced AF progression. However, although low numbers, we did not find significant differences in rhythm control therapy during follow-up between the groups. The clinical risk model obviously depends on the population included in the trial. Second, the existence of missing values in the data, which might have impacted the model, although we used multiple imputation to use the non-missing part of the data as much as possible as opposed to the removal of information from the analysis when doing regression analyses only with patients with complete information only. Third, follow-up was a median of 2.2 years, and did not met the calculated sample size of 750 patients and expected 187 AF progression events, due to a slow inclusion rate as result of the COVID-19 pandemic. To further assess AF progression, more patients and longer follow-up is needed. Fourth, for validation of the models and risk score an impact study is needed before use in clinical practice. However, according to our knowledge such a cohort with continuous rhythm monitoring and thorough phenotyping is not yet available. Fifth, not all factors contributing to AF progression may have been systematically, and repeatedly, assessed in RACE V. Finally, we did not implement temporal dynamic risk profiling into our study.^30^

## Conclusion

The RACE V study shows that AF progression in patients with PAF occurs in 5.5% per year as assessed with continuous rhythm recording. Clinical predictors for AF progression included markers of an atrial cardiomyopathy, higher waist circumference and male sex. The addition of cardiovascular biomarkers improved the risk prediction model and showed that increased levels of markers for atrial remodelling, inflammation, atherosclerosis and coagulation were predictive for AF progression.

## Data Availability

Data are available on reasonable request. Data are available on request.
