# Supplementary material for: Prevalence and determinants of atrial fibrillation progression in paroxysmal atrial fibrillation
Source: Heart. 2022 Jul 20;109(3):186–94. doi: 10.1136/heartjnl-2022-321027 (PMC9872250; doi:10.1136/heartjnl-2022-321027)
Supplement: Supplementary data [file heartjnl-2022-321027supp001.pdf]

Supplementary Data

Prevalence and determinants of AF progression in self-terminating atrial fibrillation - data from RACE V.

Bao-Oanh Nguyen MD; Vanessa Weberndorfer, MD; Harry J.G.M. Crijns, MD, PhD; Bastiaan Geelhoed, PhD; Hugo ten Cate, MD, PhD; Henri Spronk, PhD; Abraham A. Kroon, MD, PhD; Ruben R. De With, MD; Meelad I.H. Al-Jazairi, MD; Alexander H. Maass MD, PhD; Yuri Blaauw, MD, PhD; Robert G. Tieleman, MD, PhD; Martin E.W. Hemels, MD, PhD; Justin Luermans, MD, PhD; Joris R. de Groot, MD, PhD; Cor P. Allaart, MD, PhD; Arif Elvan, MD, PhD; Mirko de Melis, PhD; Coert O.S. Scheerder, MSc; Ulrich Schotten, MD, PhD; Dominik Linz, MD, PhD; Isabelle C. Van Gelder, MD, PhD; Michiel Rienstra, MD, PhD; for the RACE V Investigators.

List of Contents

1. Core lab measurement methods page 02
2. Clinical definitions used in this study page 05
3. Extensive statistical description page 06
4. Supplementary Figure S1 RACE V study design overview page 09
5. Supplementary Figure S2. RACE V rhythm control therapy overview page 10
6. Supplementary Table S1. RACE V inclusion and exclusion criteria page 11
7. Supplementary Table S2. List of 92 biomarkers, Olink Cardiovascular III panel (v.6113) page 12
8. Supplementary Table S3. Inclusion distribution per participating centre page 15
9. Supplementary Table S4. Baseline Characteristics of AF progression groups page 16
10. Supplementary Table S5. Olink biomarkers at baseline page 19
11. Supplementary Table S6. Coagulation markers page 22
12. Supplementary Table S7. Age and sex adjusted of clinical factors related to AF progression page 23
13. Supplementary Table S8. Age and sex adjusted analysis with biomarkers page 24

Nguyen B-O, *et al. Heart* 2023; 109:186–194. doi: 10.1136/heartjnl-2022-321027

Core lab measurement methods

Reveal LINQ and pacemaker arrhythmia episode adjustments

All collected episodes continuous data on arrhythmias of the included patients up until 1 May 2020 saved by the Reveal LINQ and pacemaker were independently adjudicated and corrected by 5 physicians. Any episode of AF ≥2 minutes was automatically detected. Arrhythmias with ≥182 beats per minute (cycle length ≤330 ms) with a duration of ≥24 beats were automatically classified as tachycardia. Arrhythmias with ≤30 beats per minute (cycle length ≥2000 ms), lasting 12 beats were automatically classified as bradycardia. An asystole ≥4.5 seconds was classified as a pause. The most common reasons for adjustments were false positive AF episodes (premature atrial or ventricular complexes or artefacts), on-going episodes, and episodes classified as atrial tachycardia instead of AF.

Echocardiography

Echocardiography recordings were anonymized and transferred to a core-lab facility for further analysis. Strain analysis was conducted offline, during one cardiac cycle, in sinus rhythm by one experienced observer blinded to clinical data and outcomes. Analysis was performed using vendor-independent software (TOMTEC-ARENA, Imaging Systems, Germany). LV global longitudinal strain (GLS) was analysed in the apical two-, three- and four-chamber views. Left atrial, right atrial and right ventricular strain were assessed in apical four-chamber view only. The region of interest was determined by a manual point-and-click method to trace endocardial borders during LV end-systolic frame. End-systole was automatically defined by the software and was manually adjusted for accuracy when needed. The software automatically produced myocardial speckle tracking in each frame during one cardiac cycle (RR-interval). Atrial contractile function measurements were performed by setting the base or zero strain reference at left ventricular end-diastole. Therefore, left atrial (LA) reservoir strain was measured as difference of the strain value at mitral valve opening minus the zero strain reference. LA contractile function was measured as the difference of the peak strain value at the onset of atrial contraction minus the zero strain reference.

Nguyen B-O, *et al. Heart* 2023; 109:186–194. doi: 10.1136/heartjnl-2022-321027

LA conduit was measured as the difference of the strain value at mitral valve opening (LA reservoir) minus the peak strain value at the onset of atrial contraction.

1. Badano LP, Kolias TJ, Muraru D, et al. Standardization of left atrial, right ventricular, and right atrial deformation imaging using two-dimensional speckle tracking echocardiography: A consensus document of the EACVI/ASE/industry task force to standardize deformation imaging. *Eur Heart J Cardiovasc Imaging*. 2018;19(6):591-600.

Vascular assessment

Vascular assessment of the carotid arteries included measurements of intima media thickness (IMT), pulse wave velocity (PWV) and plaques. PWV was assessed by Complior (Alam Medical, France) or SphygmoCor (Atcor Medical Blood Pressure Analysis System, Australia) at the carotid and femoral arteries. The aortic PWV was determined by using ≥ 20 consecutive pressure waveforms at the carotid and femoral artery. The wave transit time was calculated by the system software using the R-wave from the simultaneous ECG recording. of the simultaneously recorded ECG. Distance between both measure points was determined and corrected by multiplying the distance by 0.8. The PWV was calculated by dividing the distance between the femoral and carotid artery by the wave transit time. IMT and presence of plaques was assessed by ultrasound (Siemens Acuson S2000) with the Syncho US Workplace 3.5, Arterial Health Package for automated IMT measurement. Assessment of the IMT was done bilaterally in the common carotid artery, the carotid bifurcation, and internal carotid artery.

Cardiac computed tomography (CT)

Epicardial fat was measured on ECG-triggered, native CT heart scans according to the methodology introduced by Fox et al.(1). Tube voltage of scan protocols varied between 80–120kV. The region of interest (ROI) was defined as described by Versteylen(2): The cranial slice limit was set at the level of the

carina of the pulmonary artery, and the caudal slice limit was the last slice containing any portion of the heart. The anterior border of the ROI was defined by the sternum, the posterior border by the ribs and vertebral column. Images were reconstructed using a soft-tissue algorithm. The pericardium was traced by a blinded reader placing 5-7 control points per slice using axial views as described earlier. Afterwards Catmull-Rom cubic spline functions are then automatically generated to obtain a smooth closed pericardial contour. Ultimately fat was automatically summed with a dedicated volumetric software (syngo.via Frontier, Cardiac risk assessment package, Siemens Healthineers, Forchheim, Germany). Epicardial and pericardial fat were defined as previously described by Iacobellis: Epicardial fat is located between the outer wall of the pericardium and the visceral layer of the pericardium. Pericardial fat is localized between visceral and pericardial myocardium(3).

1. Fox CS, Gona P, Hoffmann U, Porter SA, Salton CJ, Massaro JM, et al. Pericardial fat, intrathoracic fat, and measures of left ventricular structure and function: the Framingham Heart Study. Circulation. 2009;119(12):1586-91.
2. Versteylen MO, Takx RA, Joosen IA, Nelemans PJ, Das M, Crijns HJ, et al. Epicardial adipose tissue volume as a predictor for coronary artery disease in diabetic, impaired fasting glucose, and non-diabetic patients presenting with chest pain. Eur Heart J Cardiovasc Imaging. 2012;13(6):517-23.
3. Iacobellis G. Epicardial and pericardial fat: close, but very different. Obesity (Silver Spring). 2009;17(4):625; author reply 6-7.

Blood biomarkers

At baseline peripheral blood samples were collected. Patients needed to be in sinus rhythm during blood sampling and oral anticoagulation was temporarily interrupted. All blood samples were processed and stored at -80°C.

Clinical definitions used in this study

Heart Failure definition

At baseline presence of:
1) history of heart failure admission;
2) left ventricular ejection fraction ≤45%;
3) left ventricular ejection fraction >45%,

with either signs of:

- structural heart disease (left ventricular hypertrophy [left ventricular mass index >95 g/m^2 in women and >115 g/m^2 in men] OR posterior wall thickness ≥11 mm OR septal wall thickness ≥11 mm)
- and/or signs of diastolic dysfunction (mean E' velocity <8 cm/s & deceleration time >220ms & E/e' >8).

Heart Failure with Preserved Ejection Fraction (HFpEF) definition

- combination of LVEF >45% + structural heart disease

and/or

- combination of LVEF >45% + diastolic dysfunction

Hypertension definition

At baseline presence of:

- History of hypertension
- Use of a beta blocker, with exception of not daily used.
- Use of any calcium channel blocker
- Use of any ACE-inhibitor
- Use of any angiotensin receptor blocker
- Use of any diuretic, including mineralocorticoid receptor antagonist, excluding furosemide amiloride and bumetanide use.
- Use of an alpha blocker
- Baseline blood pressure >140/90mmHg.

Atherosclerosis definition

At baseline presence of:

- history of myocardial infarction
- history of percutaneous coronary intervention
- history of coronary artery bypass graft
- history of ischemic cerebral infarction
- history of peripheral vascular disease
- coronary Agatston score of >400
- plaque on vascular measurement

Nguyen B-O, *et al. Heart* 2023; 109:186–194. doi: 10.1136/heartjnl-2022-321027

Extensive statistical description

Fisher's exact was used for binary variables and the T-test and Wilcoxon test were used depending on normality for continuous variables. For non-binary categorical variables, the Chi-squared test with simulation

Multivariable logistic regression model
Collected baseline variables including core lab data, with p<0.10 in the age and sex adjusted logistic regression, with exception of EHRA class, number of comorbidities, CHA_2DS_2-VASc score and medications, were included in a bidirectional step-wise variable selection, starting with age and sex in the model. Variables were then added to the model in order of (increasing) p-value of age and sex-adjusted analyses, starting with the variable that has the lowest p-value in the age and sex-adjusted logistic regressions.
Before a new variable is added, variables in the model with p>=0.05 were identified and removed, starting with the one with the biggest p-value. Before each potential next removal, the model was refit and thus the recalculated p-value was used to determine if there was a next variable with p>=0.05. If no variables were to be removed the next variable was added.
The bi-directional stepping consists of a single forward stepping of all the variables, interspersed with backwards stepping of the variables in the model before each next step in the forward-stepping.
The statistical criterion for removing a variable from the model (during each backwards stepping) was p>=0.05.
The step-wise variable selection will ensure that no variables with p>=0.05 will end up in the final multivariable model. However, due to possible negative confounding even a variable with p>=0.05 in an age and sex-adjusted model, may have p<0.05 in a model with additional covariates. The aim was also not to keep a too big set of variables to be included in the step-wise process. Therefore, a p<0.10 was selected as a trade-off between the most stringent selection (based on p<0.05) and the least-stringent selection (that is without taking into account the p-value of age and sex-adjusted regressions).
In the final multivariate model (obtained at the end of the bidirectional stepping), testing for each possible second-order interaction was done (i.e. an interaction between two variables or an interaction with itself (quadratic term), what happened if this interaction is added to the model). Specifically, the p-value of the interaction was checked. Of all possible interactions, none reached Bonferroni significance (taking into account multiple testing).

Hosmer and Lemeshow test was used for goodness of fit test with 8 degrees of freedom, Chi-squared = 6.68, p-value = 0.572
Discrimination slope of the main model = 0.082

Imputation
Imputation was implemented for missing values using the R package mice. Mice creates multiple imputations for multivariate missing data. For this article 4000 imputations for each model fit that required imputation were performed. Each incomplete variable was imputed by a separate model. The default methods of predictive mean matching for numeric data and logistic regression for binary data.
For each logistic regression "massive imputation" was performed, which means that all variables in a model were at the same time also used for the imputation needed for the fit of that model. Internally, mice performs the logistic regression fit on all 4000 imputations. It pools the results according to Rubin's rules for imputation, with a small sample refinement of the method to compute degrees of freedom according to Barnard and Rubin.

1. van Buuren,Stef, Groothuis-Oudshoorn,Karin. Mice: Multivariate imputation by chained equations in R. *Journal of Statistical Software*. 2011;45(3):1-67.

Internal validation

Internal validation was accessed using bootstrapping.(1) Fifty bootstrap samples were used.(2). The optimism caused by overfitting in the C-statistic of our model without biomarkers to be 3.03%."

1. Moons KGM, Kengne AP, Woodward M*, et al*
Risk prediction models: I. Development, internal validation, and assessing the incremental value of a new (bio)marker *Heart* 2012;**98:**683-690.
2. Fernandez-Felix BM, García-Esquinas E, Muriel A, Royuela A, Zamora J. Bootstrap internal validation command for predictive logistic regression models. *The Stata Journal*. 2021;21(2):498-509.

Risk score

The multivariable model was used to calculate the linear predictor for all patients with complete data. This was done in the standard way, namely linear combinations of the variables with the beta coefficients as weights, specifically we obtained the following expression for the linear predictor (centering continuous variables on their respective means):
Linear prediction.= Female sex * (-0.5586208) + (PR-interval - 168.8658) * 0.01309933 + (LA contraction function - 17.30064) * (-0.08169752) + (waist circumference - 101.1294) * 0.02787357 + mitral valve regurgitation * 1.771005
Next, these linear predictors was used to calculate a factor (called F here for simplicity), such that the 95% interval of the linear predictors (from the 2.5% quantile to the 97.5% quantile), when multiplied with this factor, is of length 10. This was done, because the aim was a point-based risk score that can vary from 0 up to and including 10 with only very few occurrences outside of this interval.

F can be interpreted as a conversion factor such that the product of a variable with its beta coefficient and F represents a certain number of points. This factor F was then used to obtain a preliminary scoring scheme in the following way:
For binary variables, the no-level gets zero points and the yes-level gets beta * F points. The number of points was rounded to the nearest integer value. For continuous variables, the step size is defined as the inverse of the absolute value of its beta coefficient and F rounded up to the nearest integer value. Step size can be interpreted as the number of units of the variable per point. The number of levels the variable has in the point-based risk score is then set to the range of the variable (maximum minus minimum) divided by the step size rounded down to the nearest integer value.
The range of the variable is then divided using intervals of length equal to the step size and centered in the range of the variable. The number of points assigned to the interval is the value of the variable in its midpoint times its beta coefficient times F, rounded to the nearest integer value. Finally, the first interval is the extended to include also the smallest values of the variable and the last one to include also the largest values, so that the entire range of the variable is covered. Age was removed from the point based risk score.

Mathematical formula of AF progression

$B = \Sigma_i T_i$

B = AF burden
Σ_i = sum of all AF episodes in the time period of which the AF burden or weighted AF burden is calculated.
T_i = time of AF episode i.

$B_{weighed} = \Sigma_i w_i T_i$

$B_{weighed}$ = weighted AF burden,
Σ_i = sum of all AF episodes in the time period of which the AF burden or weighted AF burden is calculated.
w_i = weight factor for AF episode i.

$w_i = 2 \times (t_i - t_{start})/(t_{stop} - t_{start})$

t_i = time when AF episode took place (time choses is in the middle of the episode)
t_{start} = start time of the period of which the weighted AF burden is calculated,
t_{stop} = end time of the period of which the weighted AF burden is calculated.

T_i = length of time of AF episode i.

AF progression is calculated with the formulas of B and $B_{weighed}$:
$P = B_{weighed} - B$

P = AF progression.

Progression is presented as percentage

$100 \times (P/ (t_{stop} - t_{start}))\%$

t_{start} = start time of the period of which the weighted AF burden is calculated,
t_{stop} = end time of the period of which the weighted AF burden is calculated.

An increase >3% AF burden over the first six months or total follow-up was chosen as definition for atrial fibrillation progression. This cut-off point was chosen because the results were most consistent with the assessment of the physicians.

Supplementary Figure S1. RACE V study design overview

Investigations at baseline and during follow-up	T = 0	T = 1 year	T = 2.5 years	End of device/ End of study*
Clinical history	●	●	●	
Examination	●	●	●	
Blood	●		●	
Electrocardiogram	●	●	●	
Echocardiography	●		●	
Blood biomarkers	●		●	
Questionnaires	●	●	●	
Genotyping	●			
Vascular assessment	●			
CT heart	●			
Adverse events	●	●	●	●
Rhythm monitoring	●	●	●	●

* End of study was variable and dependent on form of consent for study follow-up with continuous rhythm monitoring until 2,5 years, at end of battery of Reveal LINQ or at 4 years for patients with a pacemaker.

Supplementary Figure S2. RACE V rhythm control therapy overview

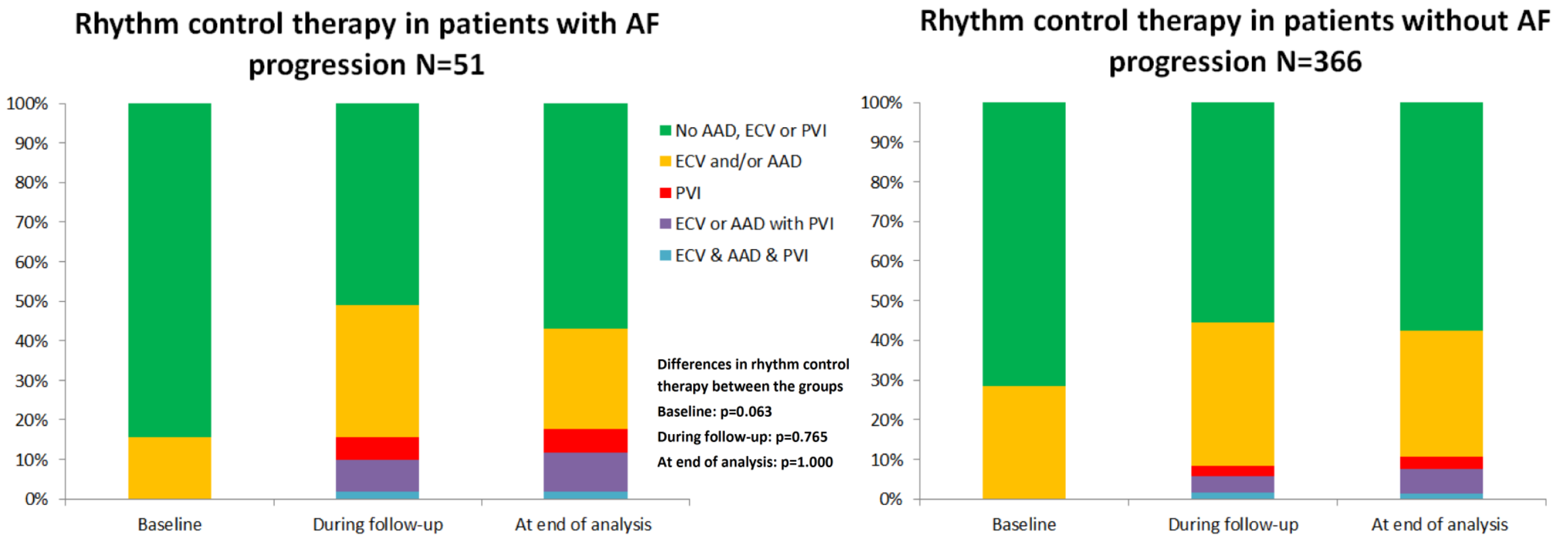

Use of rhythm control therapy.
AAD= antiarrhythmic drug; AF= atrial fibrillation; ECV = electro cardioversion; PVI = pulmonary vein isolation
2 patients with AF progression used amiodarone, both started during follow-up, 1 stopped during follow-up, 1 continued until end of analysis.
4 patients without AF progression used amiodarone, all started during follow-up, 1 stopped during follow-up, 3 continued until end of analysis.
5 of 26 patients (19%) undergoing PVI showed AF progression. 9 of 30 patients (30%) undergoing ECV showed AF progression. 3 of 14 (21%) patients undergoing both ECV and PVI showed AF progression.

Supplementary Table S1. RACE V inclusion and exclusion criteria

**Inclusion criteria**	Age > 18 years
	Total history <10 years of paroxysmal, self-terminating AF
	At least one documented episode of AF and 2 symptomatic episodes or two documented episodes, documented as:<br>- AF on ECG, Holter-recording, loop recorder, event recorder or MyDiagnostick; or<br>- Subclinical AF (SCAF) detected in a Medtronic pacemaker (atrial rate > 190 bpm lasting > 6 minutes)
	Able and willing to sign informed consent for the registry
	Able and willing to undergo implantation of ILR (in patients without a CIED)
	CHA2DS2-VASc score ≤5
	No other indication for oral anticoagulation (e.g. mechanical valve prosthesis)
**Exclusion criteria**	Non-self-terminating, persistent AF;
	Only AF due to a trigger (i.e. postoperative, due to infection)
	Congenital heart disease
	Refusing to temporarily stop (N)OAC for coagulation phenotyping (in patients already on (N)OAC before inclusion in this study), with the exception for patients with a history of ischemic stroke/ transient ischemic attack;
	Prior pulmonary vein isolation (PVI) or on waiting list for PVI or expected to be placed on waiting list within one year, since it is expected that those patients will not show much AF recurrences.
	Expected to start with, or currently using amiodarone, since it is expected that those patients will not show AF recurrences.
	Pregnancy
	ICD, CRT or pacemaker that is not a Medtronic pacemaker due to differences in AHRE algorithm or incompatibility with the type of home-monitoring
	Life expectancy of less than 2.5 years
	Ventricular pacing >50% in patients with a Medtronic pacemaker

Supplementary Table S2. List of 92 biomarkers, Olink Cardiovascular III panel (v.6113)

Abbreviations	Biomarkers	Uniprot ID	OlinkID
ALCAM	CD166 antigen	Q13740	OID00572
AP-N	Aminopeptidase N	P15144	OID00611
AXL	Tyrosine-protein kinase receptor UFO	P30530	OID00612
AZU1	Azurocidin	P20160	OID00597
BLM hydrolase	Bleomycin hydrolase	Q13867	OID00581
CASP-3	Caspase-3	P42574	OID00630
CCL15	C-C motif chemokine 15	Q16663	OID00629
CCL16	C-C motif chemokine 16	O15467	OID00654
CCL24	C-C motif chemokine 24	O00175	OID00592

CD163	Scavenger receptor cysteine-rich type 1 protein M130	Q86VB7	OID00577
CD93	Complement component C1q receptor	Q9NPY3	OID00639
CDH5	Cadherin-5	P33151	OID00587
CHI3L1	Chitinase-3-like protein 1	P36222	OID00633
CHIT1	Chitotriosidase-1	Q13231	OID00605
CNTN1	Contactin-1	Q12860	OID00586
COL1A1	Collagen alpha-1(I) chain	P02452	OID00641
CPA1	Carboxypeptidase A1	P15085	OID00624
CPB1	Carboxypeptidase B	P15086	OID00632
CSTB	Cystatin-B	P04080	OID00575
CTSD	Cathepsin D	P07339	OID00622
CTSZ	Cathepsin Z	Q9UBR2	OID00643
CXCL16	C-X-C motif chemokine 16	Q9H2A7	OID00601
DLK-1	Protein delta homolog 1	P80370	OID00598
EGFR	Epidermal growth factor receptor	P00533	OID00637
Ep-CAM	Epithelial cell adhesion molecule	P16422	OID00610
EPHB4	Ephrin type-B receptor 4	P54760	OID00569
FABP4	Fatty acid-binding protein, adipocyte	P15090	OID00589
FAS	Tumor necrosis factor receptor superfamily member 6	P25445	OID00615
Gal-3	Galectin-3	P17931	OID00578
Gal-4	Galectin-4	P56470	OID00626
GDF-15	Growth/differentiation factor 15	Q99988	OID00595
GP6	Platelet glycoprotein VI	Q9HCN6	OID05026
GRN	Granulins	P28799	OID00579
ICAM-2	Intercellular adhesion molecule 2	P13598	OID00646
IGFBP-1	Insulin-like growth factor-binding protein 1	P08833	OID00604
IGFBP-2	Insulin-like growth factor-binding protein 2	P18065	OID00650
IGFBP-7	Insulin-like growth factor-binding protein 7	Q16270	OID00638
IL-17RA	Interleukin-17 receptor A	Q96F46	OID00566
IL-18BP	Interleukin-18-binding protein	O95998	OID00640
IL-1RT1	Interleukin-1 receptor type 1	P14778	OID00613
IL-1RT2	Interleukin-1 receptor type 2	P27930	OID00627
IL-2RA	Interleukin-2 receptor subunit alpha	P01589	OID00570
IL-6RA	Interleukin-6 receptor subunit alpha	P08887	OID00602
ITGB2	Integrin beta-2	P05107	OID00565
JAM-A	Junctional adhesion molecule A	Q9Y624	OID00625
KLK6	Kallikrein-6	Q92876	OID00647

LDL receptor	Low-density lipoprotein receptor	P01130	OID00564
LTBR	Lymphotoxin-beta receptor	P36941	OID00583
MB	Myoglobin	P02144	OID00616
MCP-1	Monocyte chemotactic protein 1	P13500	OID00576
MEPE	Matrix extracellular phosphoglycoprotein	Q9NQ76	OID00132
MMP-2	Matrix metalloproteinase-2	P08253	OID00614
MMP-3	Matrix metalloproteinase-3	P08254	OID00644
MMP-9	Matrix metalloproteinase-9	P14780	OID00568
MPO	Myeloperoxidase	P05164	OID00600
Notch 3	Neurogenic locus notch homolog protein 3	Q9UM47	OID00584
NT-proBNP	N-terminal prohormone brain natriuretic peptide	NA	OID00131
OPG	Osteoprotegerin	O00300	OID00571
OPN	Osteopontin	P10451	OID00621
PAI	Plasminogen activator inhibitor 1	P05121	OID00591
PCSK9	Proprotein convertase subtilisin/kexin type 9	Q8NBP7	OID00619
PDGF subunit A	Platelet-derived growth factor subunit A	P04085	OID00648
PECAM-1	Platelet endothelial cell adhesion molecule	P16284	OID00652
PGLYRP1	Peptidoglycan recognition protein 1	O75594	OID00623
PI3	Elafin	P19957	OID00609
PLC	Perlecan	P98160	OID00582
PON3	Paraoxonase	Q15166	OID00642
PRTN3	Myeloblastin	P24158	OID00618
PSP-D	Pulmonary surfactant-associated protein D	P35247	OID00608
RARRES2	Retinoic acid receptor responder protein 2	Q99969	OID00645
RETN	Resistin	Q9HD89	OID00603
SCGB3A2	Secretoglobin family 3A member 2	Q96PL1	OID00636
SELE	E-selectin	P16581	OID00596
SELP	P-selectin	P16109	OID00574
SHPS-1	Tyrosine-protein phosphatase non-receptor type substrate 1	P78324	OID00628
SPON1	Spondin-1	Q9HCB6	OID00599
ST2	ST2 protein	Q01638	OID00634
TFF3	Trefoil factor 3	Q07654	OID00573
TFPI	Tissue factor pathway inhibitor	P10646	OID00590
TIMP4	Metalloproteinase inhibitor 4	Q99727	OID00585
TLT-2	Trem-like transcript 2 protein	Q5T2D2	OID00588
TNF-R1	Tumor necrosis factor receptor 1	P19438	OID00649

TNF-R2	Tumor necrosis factor receptor 2	P20333	OID00567
TNFRSF10C	Tumor necrosis factor receptor superfamily member 10C	O14798	OID00594
TNFRSF14	Tumor necrosis factor receptor superfamily member 14	Q92956	OID00563
TNFSF13B	Tumor necrosis factor ligand superfamily member 13B	Q9Y275	OID00617
t-PA	Tissue-type plasminogen activator	P00750	OID00635
TR	Transferrin receptor protein 1	P02786	OID00593
TR-AP	Tartrate-resistant acid phosphatase type 5	P13686	OID00606
uPA	Urokinase-type plasminogen activator	P00749	OID00631
U-PAR	Urokinase plasminogen activator surface receptor	Q03405	OID00620
vWF	Von Willebrand factor	P04275	OID00651

Supplementary Table S3. Inclusion distribution per participating centre

**Centre**	**Number of inclusion**
University Medical Centre Groningen	100
Maastricht University Medical Centre	106
Ommelander Hospital Groningen	31
Martini Hospital	111
Rijnstate Hospital	40
University of Amsterdam	22
Isala Hospital	4
Laurentius Hospital	2
VU Medical Centre Amsterdam	2

Nguyen B-O, *et al. Heart* 2023; 109:186–194. doi: 10.1136/heartjnl-2022-321027

Supplementary Table S4. Baseline Characteristics of AF progression groups

Characteristic	No AF recurrence (group 1) (N=48)	AF recurrence without AF progression (group 2) (N=318)	AF progression without persistent AF (group 3) (N=16)	AF progression with persistent AF (group 4) (N=35)
**Age (years)**	63 (54-72)	65 (58-71)	63 (58-71)	65 (62-74)
**Female sex**	19 (40%)	145 (46%)	5 (31%)	10 (29%)
**Total history AF (years)**	1.6 (0.5-4.7)	2.6 (0.8-5.3)	2.5 (1.0-3.4)	3.6 (0.9-5.7)
**Heart failure**	11 (39%)	93 (50%)	6 (55%)	15 (63%)
**HFrEF**	2 (4%)	4 (1%)	1 (1 %)	3 (9%)
**HFpEF**	9 (19%)	89 (28%)	5 (31%)	11 (31%)
**Hypertension**	42 (88%)	250 (78%)	16 (100%)	30 (86%)
**Diabetes mellitus**	3 (6%)	26 (8%)	1 (6%)	4 (11%)
**Coronary artery disease**	4 (8%)	33 (10%)	2 (13%)	9 (26%)
**Atherosclerosis***	25 (52%)	153 (48%)	9 (56%)	17 (49%)
**Peripheral artery disease**	0 (0%)	1 (0%)	0 (0%)	2 (6%)
**Ischemic stroke**	3 (6%)	15 (5%)	1 (6%)	0 (0%)

**Chronic obstructive pulmonary disease**	0 (0%)	19 (6%)	2 (13%)	2 (6%)
**Number of Comorbidities****	2 (2-3)	2 (2-3)	3 (2-3)	3 (2-4)
**CHA_2DS_2-VASc score*****				
**≤ 2**	35 (73%)	230 (72%)	15 (94%)	30 (86%)
**>2**	13 (27%)	88 (28%)	1 (6%)	5 (14%)
**EHRA class**				
I	9 (19%)	23 (7 %)	4 (25%)	7 (20%)
IIa	16 (33%)	98 (31%)	4 (25%)	17 (49%)
IIb	16 (33%)	139 (44%)	6 (38%)	6 (17%)
III	7 (15%)	56 (18%)	2 (13%)	5 (14%)
IV	0 (0%)	2 (1%)	0 (0%)	0 (0%)
**Physical examination**				
**Height (cm)**	178 (172-183)	176 (168-184)	177 (172-187)	179 (170-183)
**Weight (kg)**	88 (77-98)	84 (74-96)	91 (72-104)	88 (75-100)
**BMI (kg/m^2)**	27(25- 30)	27 (24-30)	26 (25-30)	27 (24-32)
**Obesity (BMI>30)**	13 (27%)	79 (25%)	4 (25%)	11 (32%)
**Waist circumference (cm)**	99 (93-109)	100 (92-108)	103 (95-111)	106 (101-114)
**Systolic blood pressure (mmHg)**	133 (124-140)	134 (125-145)	130 (122-136)	130 (124-144)
**Diastolic blood pressure (mmHg)**	80 (75-85)	80 (74-85)	75 (72-83)	80 (72-85)
**Laboratory results**				
**Creatinine (µmol/L)**	77 (67-85)	80 (69-91)	89 (80-99)	87 (80-100)
**eGFR (mL/min*1.73m^2)**	85 (76-93)	81 (69-90)	71 (63-83)	76 (68-86)
**Electrocardiogram**				
**PR-interval**	166 (148-173)	164 (149-186)	170 (160-200)	180 (168-199)
**QRS-interval**	94 (88-101)	94 (86-104)	94 (90-103)	96 (90-110)
**Medications**				
**β-blocker**	25 (52%)	156 (49%)	11 (69%)	21 (60%)
**Verapamil/Diltiazem**	8 (17%)	58 (18%)	3 (19%)	4 (11%)
**Digoxin**	0 (0%)	4 (1 %)	0 (0 %)	2 (6%)
**Class I antiarrhythmic drugs**	13 (27 %)	76 (24%)	3 (19%)	2 (6%)
**Class III antiarrhythmic drugs**	1 (2%)	14 (4%)	1 (6%)	3 (9%)
**ACE-inhibitor**	12 (25%)	59 (19%)	3 (19%)	8 (23%)
**Angiotensin Receptor Blocker**	8 (17%)	58 (18%)	5 (31 %)	9 (26%)
**Statin**	10 (21%)	109 (34%)	10 (63%)	16 (46%)
**Diuretic**	7 (15%)	47 (15%)	3 (19%)	7 (20%)
**Anticoagulant**	29 (60%)	215 (68%)	13 (81%)	32 (91%)
**Vitamin K antagonist**	4 (8%)	41 (13%)	3 (19%)	7 (20%)
**NOAC**	25 (52%)	174 (55%)	10 (63%)	25 (71%)
**Echocardiographic variables** [a]				
**Left atrial volume (mL)**	58 (46-67)	58 (47-74)	60 (55-76)	68 (55-85)
**Left atrial volume index (mL/m^2)**	28 (22-34)	29 (24-36)	32 (25-35)	35 (26-39)
**Left atrial reservoir function (%)**	38 (31-47)	37 (29-43)	35 (27- 53)	31 (25-35)

**Left atrial contractile function (%)**	18 (13-23)	17 (13-21)	15 (10-23)	13 (11-15)
**Left atrial conduction function (%)**	19 (15-24)	19 (14-24)	23 (15-34)	18 (13-23)
**Right atrial volume (mL)**	39 (34-56)	48 (38-65)	55 (47-79)	55 (46-63)
**Right atrial volume indexed (mL/m^2)**	21 (16- 29)	24 (20 - 31)	32 (23- 33)	28 (24 - 33)
**Left ventricular ejection fraction (%)**	51 ± 10	51 ± 8	52 ± 9	50 ± 8
**Left ventricular mass (g)**	150 (140-165)	148 (126-178)	152 (139-182)	161 (134-188)
**Left ventricular mass index (g/m^2)**	75 (68-83)	76 (64-88)	86 (69-88)	78 (67-96)
**Left ventricle strain**	-14.5 ± 2.5	-14.0 ± 2.3	-14.2 ± 2.6	-14.3 ± 2.6
**Computed Tomography [b]**				
**Calcium score (Agatston)**	15 (0-75)	22 (0-227)	94 (15-3270)	152 (4-917)
**Agatston >400**				
**Pericardial fat**	171 (134-223)	166 (118- 232)	205 (160-224)	167 (137-235)
**Epicardial fat**	102 (74-132)	97 (70-128)	104 (90-126)	105 (72-137)
**Vascular assessment [c]**				
**IMT max–CCA (mm)**	0.90 (0.82-1.04)	0.92 (0.81-1.07)	1.03 (0.85-1.19)	0.97 (0.83-1.13)
**IMT max-CCA**				
**>1mm**	14 (33%)	95 (34%)	8 (53%)	11 (42%)
**IMT max–all segments (mm)**	1.04 (0.93-1.20)	0.98 (0.84-1.16)	1.00 (0.88-1.20)	1.02 (0.88-1.14)
**IMT max-all segments**				
**>1mm**	24 (57%)	130 (47%)	7 (47%)	13 (50%)
**Pulse wave velocity (m/s)**	8.60 (6.98-10.00)	8.49 (7.45-10.20)	8.46 (7.66-10.25)	9.20 (8.14-10.26)
**Plaques**	23 (64%)	102 (48%)	6 (60%)	9 (60%)
**Plaques >3**	2 (4%)	10 (3%)	2 (12%)	3 (9%)

Data are presented as mean±standard deviation, number of patients (%), or median (interquartile range). Abbreviations: ACE=angiotensin-converting enzyme; AF=atrial fibrillation; BMI=body mass index; CCA= common carotid artery; eGFR=estimated glomerular filtration rate; EHRA= European Heart Rhythm Association class for symptoms; HFpEF= heart failure with preserved ejection fraction; HFrEF= heart failure with reduced ejection fraction; IMT=intima media thickness; NOAC= novel oral anticoagulation; NT-proBNP=N-terminal pro-brain natriuretic peptide; *Atherosclerosis is presence of history of myocardial infarction, percutaneous coronary intervention, coronary artery bypass graft, ischemic cerebral infarction, peripheral vascular disease, Agatston score >400 or plaque; **The number of comorbidities was calculated by awarding points for hypertension, heart failure, age >65 years, diabetes mellitus; coronary artery disease, BMI>25kg/m2, moderate or severe mitral valve regurgitation and kidney dysfunction (eGFR<60); ***The CHA2DS2-VASc score assesses thromboembolic risk. C=congestive heart failure/LV dysfunction, H=hypertension; A2=age ≥75 years; D=diabetes mellitus; S2=stroke/transient ischemic attack/systemic embolism; V=vascular disease; A=age 65-74 years; Sc=sex category (female sex).). [a]Left atrial and ventricle strain measurements could not be performed in 75 patients. Measurements of right atrial strain could not be done in 123 patients. [b]Agatston score was not available for 10 patients, epicardial and pericardial fat could not be analysed for 21 patients. [c]IMT CCA was not available for 55 patients, IMT all segments for le for 56 patients and pulse wave velocity could not be measured in 78 patients and amount of plaques could not be measured in 145 patients.

Supplementary Table S5. Olink biomarkers at baseline

Characteristic	AF progression (N=51)	No AF progression (N=366)	Total (N=417)	*P*-value
ALCAM	6.06 (5.82 - 6.20)	6.07 (5.84 - 6.22)	6.07 (5.84 - 6.22)	0.772
AP-N	5.46 (5.26 - 5.69)	5.53 (5.33 - 5.76)	5.53 (5.33 - 5.76)	0.107
AXL	9.44 (9.24 - 9.63)	9.39 (9.16 - 9.60)	9.39 (9.16 - 9.60)	0.168
AZU1	4.59 (4.33 - 5.16)	4.61 (4.28 - 5.03)	4.61 (4.28 - 5.03)	0.298
BLM hydrolase	6.26 (6.02 - 6.56)	6.21 (5.91 - 6.48)	6.21 (5.91 - 6.48)	0.181
CASP-3	8.32 (7.56 - 9.40)	8.52 (7.31 - 9.49)	8.45 (7.36 - 9.49)	0.883
CCL15	8.08 (7.90 - 8.38)	8.04 (7.78 - 8.36)	8.04 (7.78 - 8.36)	0.259
CCL16	7.45 (7.09 - 7.71)	7.44 (7.07 - 7.71)	7.44 (7.07 - 7.71)	0.929
CCL24	6.37 (5.46 - 6.88)	6.24 (5.64 - 6.87)	6.24 (5.63 - 6.87)	0.926
CD163	8.02 (7.65 - 8.39)	7.96 (7.60 - 8.25)	7.96 (7.61 - 8.27)	0.323
CD93	11.82 (11.57 - 12.00)	11.79 (11.56 -12.00)	11.79 (11.56 - 12.00)	0.690
CDH5	4.34 (4.06 - 4.59)	4.38 (4.08 - 4.59)	4.38 (4.08 - 4.59)	0.849
CHI3L1	7.46 (6.87 - 7.95)	7.22 (6.68 - 7.89)	7.22 (6.68 - 7.89)	0.128
CHIT1	7.29 (6.69 - 8.12)	7.16 (6.46 - 7.75)	7.16 (6.46 - 7.75)	0.281
CNTN1	4.75 (4.40 - 4.89)	4.78 (4.47 - 5.04)	4.78 (4.45 - 5.03)	0.161
COL1A1	3.45 (3.12 - 3.71)	3.47 (3.20 - 3.71)	3.46 (3.20 - 3.71)	0.446
CPA1	6.67 (6.34 - 7.20)	6.62 (6.21 - 7.02)	6.62 (6.21 - 7.02)	0.379
CPB1	6.61 (6.26 - 7.07)	6.53 (6.08 - 6.88)	6.53 (6.08 - 6.88)	0.130
CSTB	5.54 (5.19 - 5.80)	5.38 (5.05 - 5.76)	5.38 (5.05 - 5.76)	0.176
CTSD	5.22 (5.06 - 5.67)	5.11 (5.06 - 5.40)	5.11(5.06- 5.40)	0.021
CTSZ	5.97 (5.68 - 6.20)	5.91 (5.68 - 6.10)	5.92 (5.68 - 6.11)	0.259
CXCL16	5.85 (5.71 - 6.04)	5.90 (5.66 - 6.12)	5.90 (5.67 - 6.12)	0.489
DLK-1	7.01 (6.71 - 7.34)	6.95 (6.58 - 7.35)	6.95 (6.59 - 7.35)	0.435
EGFR	3.52 (3.38 - 3.75)	3.62 (3.45 - 3.82)	3.62 (3.45 - 3.82)	0.060
Ep-CAM	6.40 (5.91 - 7.39)	6.66 (5.91 - 7.50)	6.66 (5.91 - 7.50)	0.382
EPHB4	5.18 (4.89 - 5.31)	5.12 (4.89 - 5.32)	5.12 (4.89 - 5.32)	0.501
FABP4	6.64 (6.07 - 7.23)	6.40 (5.94 - 6.96)	6.40 (5.94 - 6.96)	0.107
FAS	6.14 (5.95 - 6.45)	6.14 (5.89 - 6.35)	6.14 (5.89 - 6.35)	0.188
Gal-3	6.61 (6.20 - 6.82)	6.51 (6.27 - 6.75)	6.51 (6.27 - 6.75)	0.281
Gal-4	4.17 (3.80 - 4.44)	4.14 (3.79 - 4.42)	4.14 (3.79 - 4.42)	0.475
GDF-15	6.60 (6.21 - 6.99)	6.38 (6.00 - 6.75)	6.38 (6.00 - 6.75)	0.010
GP6	3.10 (2.60 - 3.62)	3.12 (2.55 - 3.71)	3.13 (2.55 - 3.71)	0.904
GRN	6.92 (6.76 - 7.16)	6.93 (6.69 - 7.14)	6.93 (6.70 - 7.14)	0.331
ICAM-2	5.75 (5.42 - 5.96)	5.70 (5.38 - 5.97)	5.70 (5.39 - 5.97)	0.532
IGFBP-1	4.80 (3.99 - 5.56)	4.71 (3.82 - 5.66)	4.71 (3.82 - 5.66)	0.610
IGFBP-2	8.45 (7.82 - 8.90)	8.32 (7.65 - 8.83)	8.32 (7.65 - 8.83)	0.182
IGFBP-7	8.30 (8.07 - 8.66)	8.25 (7.99 - 8.50)	8.25 (7.99 - 8.50)	0.093
IL-17RA	4.36 (4.09 - 4.63)	4.41 (4.03 - 4.70)	4.40 (4.05 - 4.69)	0.879
IL-18BP	6.73 (6.53 - 6.99)	6.68 (6.44 - 6.95)	6.69 (6.45 - 6.96)	0.197

Nguyen B-O, *et al. Heart* 2023; 109:186–194. doi: 10.1136/heartjnl-2022-321027

IL-1RT1	6.90 (6.74 - 7.12)	6.92 (6.68 - 7.11)	6.92 (6.68 - 7.11)	0.697
IL-1RT2	5.60 (5.44 - 5.80)	5.63 (5.41 - 5.85)	5.63 (5.41 - 5.84)	0.448
IL2-RA	4.75 (4.51 - 5.14)	4.66 (4.332 - 4.93)	4.66 (4.33 - 4.92)	0.054
IL-6RA	12.82 (12.50 - 13.05)	12.85 (12.50 - 13.13)	12.84 (12.49 - 13.12)	0.634
ITGB2	6.42 (6.12 - 6.72)	6.35 (6.047 - 6.66)	6.35 (6.05 - 6.66)	0.250
JAM-A	6.82 (6.15 - 7.77)	6.95 (6.11 - 7.92)	6.95 (6.11 - 7.92)	0.738
KLK6	6.16 (5.89 - 6.40)	6.11 (5.84 - 6.33)	6.11 (5.86 - 6.34)	0.250
LDL receptor	5.42 (5.01 - 5.84)	5.52 (5.10 - 5.90)	5.52 (5.10 - 5.90)	0.385
LTBR	4.685 (4.38 - 4.98)	4.68 (4.40 - 4.91)	4.68 (4.40 - 4.92)	0.683
MB	7.36 (7.04 - 7.68)	7.23 (6.82 - 7.60)	7.23 (6.82 - 7.60)	0.060
MCP-1	4.42 (4.15 - 4.63)	4.44 (4.20 - 4.65)	4.44 (4.20 - 4.65)	0.455
MEPE	6.24 (5.86 - 6.55)	6.15 (5.88 - 6.45)	6.16 (5.87 - 6.46)	0.429
MMP-2	4.58 (4.34 - 4.80)	4.54 (4.27 - 4.79)	4.56 (4.29 - 4.79)	0.232
MMP-3	7.35 (6.82 - 7.66)	7.05 (6.58 - 7.50)	7.08 (6.59 - 7.54)	0.042
MMP-9	4.91 (4.45 - 5.30)	4.86 (4.27 - 5.48)	4.86 (4.27 - 5.48)	0.668
MPO	4.15 (3.93 - 4.56)	4.078 (3.83 - 4.37)	4.078 (3.83 - 4.37)	0.075
Notch 3	5.32 (4.88 - 5.65)	5.30 (4.95 - 5.62)	5.30 (4.95 - 5.62)	0.926
NT-proBNP	5.65 (4.75 - 6.41)	4.94 (4.02 - 5.69)	4.99 (4.09 - 5.78)	<0.001
OPG	4.570 (4.21 - 4.77)	4.50 (4.20 - 4.74)	4.50 (4.20 - 4.74)	0.488
OPN	7.00 (6.49 - 7.33)	6.82 (6.50 - 7.17)	6.82 (6.50 - 7.17)	0.211
PAI	5.70 (5.19 - 6.29)	5.78 (5.21 - 6.43)	5.77 (5.20 - 6.41)	0.714
PCSK9	3.11 (2.96 - 3.36)	3.07 (2.81 - 3.38)	3.07 (2.81 - 3.38)	0.214
PDGF subunit A	4.48 (4.00 - 4.89)	4.46 (3.87 - 5.17)	4.46 (3.87 - 5.13)	0.718
PECAM-1	5.76 (5.37 - 6.44)	5.86 (5.33 - 6.46)	5.86 (5.33 - 6.46)	0.978
PGLYRP1	8.43 (8.12 - 8.72)	8.20 (7.90 - 8.55)	8.20 (7.90 - 8.55)	<0.001
PI3	4.59(4.29 - 4.89)	4.39 (4.00 - 4.80)	4.39 (4.00 - 4.80)	0.011
PLC	7.35 (7.19 - 7.59)	7.30 (7.11 - 7.53)	7.30 (7.11 - 7.53)	0.080
PON3	5.69 (5.28 - 6.27)	6.01 (5.53 - 6.47)	6.01 (5.53 - 6.47)	0.004
PRTN3	5.62 (5.41 - 6.15)	5.50 (5.20 - 5.87)	5.50 (5.20 - 5.87)	0.019
PSP-D	3.48 (2.99 - 3.85)	3.29 (2.81 - 3.80)	3.29 (2.82 - 3.80)	0.142
RARRES2	12.19 (11.97 - 12.44)	12.26 (12.01 - 12.44)	12.26 (12.01 - 12.44)	0.477
RETN	7.03 (6.75 - 7.42)	6.876 (6.60 - 7.22)	6.88 (6.60 - 7.22)	0.013
SCGB3A2	3.19 (2.80 - 3.72)	3.15 (2.67 - 3.63)	3.15 (2.67 - 3.63)	0.756
SELE	13.30 (12.95 - 13.78)	13.23 (12.77 - 13.59)	13.24 (12.78 - 13.59)	0.324
SELP	11.11 (10.61 - 11.76)	11.10 (10.56 - 11.76)	11.10 (10.56 - 11.76)	0.799
SHPS-1	3.84 (3.63 - 4.06)	3.87 (3.66 - 4.12)	3.87 (3.65 - 4.12)	0.541
SPON1	1.21 (0.99 - 1.39)	1.15 (0.89 - 1.38)	1.146 (0.89 - 1.38)	0.152
ST2	4.97 (4.58 - 5.26)	4.94 (4.58 - 5.25)	4.94 (4.58 - 5.25)	0.888
TFF3	6.00 (5.76 - 6.25)	5.85 (5.589 - 6.08)	5.85 (5.58 - 6.08)	0.002
TFPI	10.16 (9.96 - 10.44)	10.33 (10.09 - 10.55)	10.29 (10.08 - 10.54)	0.012
TIMP4	4.25 (4.03 - 4.55)	4.17 (3.90 - 4.54)	4.20 (3.91 - 4.54)	0.248
TLT-2	5.40 (5.14 - 5.70)	5.44 (5.12 - 5.74)	5.44 (5.13 - 5.74)	0.921
TNF-R1	7.08 (6.78 - 7.32)	6.91 (6.67 - 7.16)	6.91 (6.67 - 7.16)	0.021

Nguyen B-O, *et al. Heart* 2023; 109:186–194. doi: 10.1136/heartjnl-2022-321027

TNF-R2	5.87 (5.56 - 6.11)	5.68 (5.41 - 5.96)	5.68 (5.41 - 5.96)	0.005
TNFRSF10C	7.01 (6.65 - 7.38)	6.88 (6.50 - 7.24)	6.88 (6.50 - 7.24)	0.036
TNFRSF14	5.41 (5.07 - 5.60)	5.27 (5.012 - 5.52)	5.27 (5.01 - 5.52)	0.204
TNFSF13B	7.595 (7.35 - 7.88)	7.58 (7.34 - 7.83)	7.58 (7.34 - 7.83)	0.820
t-PA	7.77 (7.40 - 8.36)	7.84 (7.26 - 8.74)	7.84 (7.26 - 8.74)	0.790
TR	5.54 (5.02 - 5.80)	5.29 (4.88 - 5.74)	5.29 (4.88 - 5.74)	0.115
TR-AP	4.73 (4.46 - 4.97)	4.81 (4.55 - 5.03)	4.79 (4.54 - 5.02)	0.136
uPA	6.33 (6.07 - 6.59)	6.30 (6.09 - 6.57)	6.31 (6.08 - 6.57)	0.873
U-PAR	5.91 (5.66 - 6.16)	5.81 (5.55 - 6.05)	5.81 (5.55 - 6.05)	0.061
vWF	7.85 (7.05 - 9.10)	7.91 (7.05 - 9.09)	7.91 (7.05 - 9.09)	0.965

Data is presented in a log2 scale in median (interquartile range).

Supplementary Table S6. Coagulation markers at baseline

Coagulation markers	Total (N=417)
Factor XIIa:C1inh (pM)	862.64 (747.28- 989.81)
Factor XIIa:antithrombin (pM)	11.28 (11.28-36.06)
Plasma Kallikrein:C1inh (nM)	0.30 (0.3-1.45)
Factor XIa:C1inh (pM)	72.04 (72.04-197.22)
Factor XIa:AT (pM)	7.90 (7.90-7.90)
Factor XIa:a1AT (pM)	56.17 (56.17-92.04)
Factor Xa:AT (pM)	421.62 (348.16- 497.15)
Factor IXa:AT (pM)	170.20 (170.20- 170.20)
Thrombin:AT (ug/L)	2.04 (2.04-3.54)

Data is presented in median (interquartile range). AT= antithrombin; a1AT= alpha-1-antitrypsin; C1inh=C1-Esterase inhibitor; nM=nanomolar; pM=picomolar

Nguyen B-O, *et al. Heart* 2023; 109:186–194. doi: 10.1136/heartjnl-2022-321027

Supplementary Table S7. Age and sex adjusted of clinical factors related to AF progression

	OR	95% CI	*P*-value
**Female sex**	0.48	0.25-0.91	0.024
**CHA_2DS_2-VASc score >1**	3.73	1.40-9.95	0.009
**Heart failure with reduced ejection fraction**	4.31	1.12-16.58	0.034
**Plaques >3**	5.54	1.59-19.36	0.008
**Peripheral artery disease**	11.98	1.05-136.78	0.046
**Waist circumference** (per SD)	1.46	1.06-2.02	0.022
**PR interval** (per SD)	1.45	1.10-1.92	0.009
**Left atrial contractile function** (per SD)	0.60	0.39-0.92	0.019
**Left atrium end diastolic volume** (per SD)	1.46	1.08-1.97	0.014
**Left atrium end diastolic volume indexed for BSA** (per SD)	1.44	1.07-1.95	0.017
**Right atrium end systolic volume** (per SD)	1.53	1.05-2.23	0.026
**Right atrium end systolic volume indexed for BSA** (per SD)	1.44	1.01-2.04	0.044

Logistic regression adjusted for age and sex. BSA= body surface area; CI=confidence interval; OR=odds ratio. CHA_2DS_2-VASc score assesses thromboembolic risk. C=congestive heart failure/LV dysfunction, H=hypertension; A_2=age ≥75 years; D=diabetes mellitus; S_2=stroke/transient ischemic attack/systemic embolism; V=vascular disease; A=age 65-74 years; Sc=sex category (female sex).

Nguyen B-O, *et al. Heart* 2023; 109:186–194. doi: 10.1136/heartjnl-2022-321027

Supplementary Table S8. Age and sex adjusted analysis including biomarkers

	OR	95% CI	*P*-value
**Female sex**	0.48	0.25-0.91	0.024
**CHA_2DS_2-VASc score >1**	3.73	1.40-9.95	0.009
**Heart failure with reduced ejection fraction**	4.31	1.12-16.58	0.034
**Plaques >3**	5.54	1.59-19.36	0.008
**Peripheral artery disease**	11.98	1.05-136.78	0.046
**Waist circumference** (per SD)	1.46	1.06-2.02	0.022
**PR interval** (per SD)	1.45	1.10-1.92	0.009
**Left atrial contractile function** (per SD)	0.60	0.39-0.92	0.019
**Left atrial end diastolic volume** (per SD)	1.46	1.08-1.97	0.014
**Left atrium end diastolic volume indexed for BSA** (per SD)	1.44	1.07-1.95	0.017
**Right atrium end systolic volume** (per SD)	1.53	1.05-2.23	0.026
**Right atrium end systolic volume indexed for BSA**(per SD)	1.44	1.01-2.04	0.044
**CTSD** (per SD)	1.32	1.01-1.73	0.043
**FABP4** (per SD)	1.47	1.06-2.05	0.021
**NTproBNP** (per SD)	2.05	1.43-2.94	<0.001
**PCSK9** (per SD)	1.37	1.03-1.81	0.030
**PGLYRP1**(per SD)	1.44	1.09-1.91	0.011
**PON3** (per SD)	0.72	0.54-0.96	0.027
**PRTN3** (per SD)	1.29	1.00-1.66	0.046
**RETN** (per SD)	1.34	1.01-1.76	0.041
**TFPI** (per SD)	0.72	0.53-0.97	0.030
**TNF-R2** (per SD)	1.49	1.11-2.01	0.009
**TNFRSF10C** (per SD)	1.43	1.03-1.98	0.033
**Factor XIIa:antithrombin** *(> median)*	0.39	0.17-0.91	0.030

**Factor XIIa:C1-esterase inhibitor** *(> median)*	0.38	0.20-0.75	0.005

Logistic regression adjusted for age and sex, with imputation. BSA= body surface area; CI=confidence interval; CTSD=capthesin D; FABP4=fatty acid binding protein 4; NTproBNP=N-terminal pro-brain natriuretic peptide; OR=odds ratio; PCSK9= Proprotein convertase subtilisin/kexin type 9; PGLYRP1= peptidoglycan recognition protein 1; PON3= paraoxonase 3; PRTN3=myeloblastin; RETN=resistin; TFPI= tissue factor pathway inhibitor; TNF-R2=tumor necrosis factor receptor 2; TNFRSF10C=tumor necrosis factor receptor superfamily member 10C. CHA_2DS_2-VASc score assesses thromboembolic risk. C=congestive heart failure/LV dysfunction, H=hypertension; A_2=age ≥75 years; D=diabetes mellitus; S_2=stroke/transient ischemic attack/systemic embolism; V=vascular disease; A=age 65-74 years; Sc=sex category (female sex).

Nguyen B-O, *et al. Heart* 2023; 109:186–194. doi: 10.1136/heartjnl-2022-321027
